# Amoebic Dysentery Complicated by Hypovolemic Shock and Sepsis in an Infant with Severe Acute Malnutrition: A Case Report

**DOI:** 10.3390/microorganisms11010165

**Published:** 2023-01-08

**Authors:** Giulia Dal Canto, Tawaddud Hassan Eisa Artaiga, Abdulrahman Ibrahiem Mohamed, Hayat Amin Makki Hassan, Doaa Mahmoud Adam, Moram Awadalla Ibrahiem Ahmed, Jihad Motwali, Manuela Valenti, Susanna Esposito

**Affiliations:** 1 Pediatric Clinic, Pietro Barilla Children’s Hospital, Department of Medicine and Surgery, University of Parma, Via Gramsci 14, 43126 Parma, Italy; 2 Emergency NGO Onlus, 20122 Milan, Italy

**Keywords:** amoebiasis, antimicrobial resistance, diarrhea, *Entamoeba histolytica*, malnutrition

## Abstract

Diarrheal disease continues to be a leading cause of death in children under five years old in developing countries, where it is responsible for the death of approximately half a million children each year. Establishing the cause of diarrheal disease can be difficult in developing areas due to the lack of diagnostic tests, and thus empirical therapies are often required. In these settings, the choice of antibiotic (or the choice to not give it) depends on suspected agents, host conditions and local epidemiology. Herein, we report a representative case of a ten-month-old male patient with severe acute malnutrition (SAM) admitted to the Emergency Paediatric Clinic in Port Sudan for amoebic dysentery complicated by hypovolemic shock and sepsis, treated by target therapy for *Entamoeba histolytica* infection associated with empiric antibiotic therapy. Due to the absence of clinical improvement, Ciprofloxacin was added to the first-line treatment. This case highlights that in low-income countries amoebiasis, especially in children with SAM, may result in life-threatening complications. Although stool microscopy remains the most used diagnostic test in these settings, a novel inexpensive, easy to use and rapid diagnostic test would be warranted to reach a microbiological diagnosis and guide clinical decision. Further studies will be necessary to identify the patterns of antimicrobial resistance in order to appropriately manage these complicated cases.

## 1. Background

Diarrheal disease continues to be a leading cause of death in children under five years old in developing countries, where it is responsible for the death of approximately half a million children each year [1]. Common etiologies of diarrheal disease in low-income countries are Rotavirus, *Shigella*, *Cryptosporidium*, *Campylobacter*, *Salmonella* and enterotoxigenic and shigatoxin-producing *Escherichia coli* (ET-STEC) [2,3]. Amoebiasis is also a widespread cause of diarrhea in several developing areas of Africa, Central and South America and Asia [4]. Infections caused by multiple agents are possible and can be facilitated by immunodeficiency conditions such as malnutrition or HIV infection.

The major goal of the treatment of diarrheal disease is to restore hydration and prevent dehydration [5]. Avoiding fasting and continuing feeding are also important to reduce morbidity. The routine use of antimicrobials for diarrhea in children is not recommended by the World Gastroenterology Organization (WGO) except for clinically recognizable severe cases and in the following circumstances: cholera, shigellosis, typhoid and paratyphoid fever, dysenteric presentation of campylobacteriosis and non-typhoidal salmonellosis (NTS) when they cause persistent diarrhea, invasive intestinal amebiasis, symptomatic giardiasis and when host immune status is compromised for any reason including severe malnutrition, chronic disease and lymphoproliferative disorders [6]. Antimicrobial treatment should also be considered in diarrhea with fever and/or with bloody stools and moderate–severe traveler’s diarrhea [6]. The use of antibiotics outside of these indications is discouraged for the following reasons: (1) most episodes of diarrhea are viral; (2) increasing prevalence of antimicrobial resistance has been reported and the unnecessary use of antibiotics should be avoided; (3) in some circumstances, antibiotics may worsen the outcome, i.e., in shigatoxin-producing *Escherichia coli* (STEC) infection, they may increase the risk of hemolytic uremic syndrome and in NTS, they may prolong excretion [3]. Nevertheless, establishing the cause of diarrheal disease can be difficult in developing areas due to the lack of diagnostic tests, and thus empirical therapies are often required. In these settings, the choice of antibiotic (or the choice to not give it) depends on suspected agents, host conditions and local epidemiology. However, observational studies are lacking and the indication for empiric antibiotic therapy are not standardized.

Herein, we report a representative case of an infant with severe acute malnutrition (SAM), admitted to the Emergency Paediatric Clinic in Port Sudan for amoebic dysentery complicated by hypovolemic shock and sepsis, and treated by target therapy for Entamoeba histolytica infection associated with empiric antibiotic therapy.

## 2. Case Presentation

A ten-month-old male patient from Sudan presented to the Emergency Paediatric Clinic in Port Sudan with a history of diarrhea, vomiting and fever from 3 days. On arrival at our hospital, the child appeared unwell; he was febrile (38.5 °C), tachycardic (153 bpm), with other vital signs within the acceptable limits according to age. He had severe acute malnutrition according to the WHO weight-for-height chart (W/H < −3SD). He was lethargic, with capillary refill > 3 s, cold extremities, weak peripheral pulses, slow skin pinch and sunken eyes. He had tender and distended abdomen and crepitations at the chest. As the child was showing signs of hypovolemic shock and sepsis, he received in the emergency room a bolus of 1/2 normal saline + 1/2 dextrose 5% 10 mL/kg iv in 1 h, O_2_ 0.5 L/min on nasal cannula and wide spectrum antibiotic therapy with Ampicillin 50 mg/kg TID iv and Gentamicin 7.5 mg/kg OD iv. After the first stabilization and treatment, the child was admitted to our pediatric ward.

At the clinical reassessment, he still presented signs of severe dehydration and he received a second bolus iv (1/2 normal saline + 1/2 dextrose 5% 10 mL/kg in 1 h), followed by 10 h of alternated oral ReSoMal 10 mL/kg and F75, with improvement of hydration. Blood test revealed leukocytosis (white blood cells [WBC] 32,900/µL, granulocytes 19,200/µL), metabolic acidosis (pH 7.31, PaCO_2_ 16 mmHg, Lactate 1.2 mmol/L, HCO_3_—8.1 mmol/L, BE—18.2 mmol/L) and hypokalemia (K 2.7 mmol/L). The urine microscope examination, HIV rapid test, malaria rapid test and blood film were negative. A stool microscope examination showed uncountable leukocytes, abundant mucous and few red blood cells associated with the presence of *Entamoeba histolytica* parasites. Due to these findings, the child was started on Metronidazole 10 mg/kg TID iv and Ceftriaxone 100 mg/kg OD iv and Ampicillin and Gentamicin were stopped. Hypokalemia was treated with oral integration of Potassium chloride 1 mEq/kg two times, with subsequent normalization of the values on day 3. On admission, Omeprazole iv was also started to avoid stress-induced gastritis.

During the following 48 h, the child had persistent diarrhea, vomits and intermittent fever. On day 3 from admission, he again developed signs of severe dehydration and he received one bolus of 1/2 normal saline + 1/2 dextrose 5% 10 mL/kg iv in 1 h and a subsequent blood transfusion of whole blood 10 mL/kg, with clinical improvement. Considered the persistence of symptoms despite the antibiotic therapy, Ciprofloxacin 15 mg/kg BID iv was added to the treatment for 5 days (day 3).

The clinical condition of the child gradually improved, and he maintained stable vital signs and apyrexia. Diarrhea and vomits reduced and the child went through the transition and rehabilitation phase of re-feeding, gaining body weight (+12% in 7 days). At discharge, his WBC had decreased and the granulocyte were normalized. The stool microscope examination became negative for *Entamoeba histolytica*. Table 1 summarizes the blood examinations at admission and during hospitalization.

The patient was discharged with instructions to complete antibiotic therapy with oral metronidazole and ciprofloxacin at home (for a total duration of treatment of 7 days). We also referred the child to the local feeding center for malnutrition for follow up.

## 3. Discussion

Amoebiasis is a public health problem, especially among rural areas of developing countries. In the large Global Enteric Multicenter Study (GEMS), amebiasis ranked among the top seven pathogens causing dysentery in children under the age of five years living in seven countries of sub-Saharan Africa and South Asia [2]. Amoebic disease is caused by the protozoan parasite *Entamoeba histolytica*. Infection usually begins with the ingestion of the cysts in water or food that has been contaminated by human feces. The parasite travels through the stomach and the small intestine, and excysts within the terminal ileum or colon to form the trophozoite stage. Trophozoites reproduce by binary fission and encyst, completing the lifecycle when infectious cysts are excreted into the environment in stool. Secreting proteinases that dissolve host tissues, *Entamoeba histolytica* trophozoites invade the intestinal mucosa, causing amoebic colitis and dysentery [7]. In some cases, amoebas can travel through the portal circulation to the liver, where they cause abscesses. Amoebic dysentery also may progress to fulminant colitis, toxic megacolon, colonic ulcers and perforation [8]. Furthermore, intestinal amebiasis can manifest as asymptomatic colonization or non-invasive recurrent diarrhea, which may adversely affect growth and nutritional status [9].

In the present case, the diagnosis of amoebic dysentery was reached with stool microscope examination. In areas where amoebiasis is endemic, as in our pediatric clinic in Port Sudan, amoebic colitis is commonly diagnosed by identifying cysts or motile trophozoites in the stool microscopy. However, this method has two main limitations: its accuracy is highly dependent on the competence of the diagnostic laboratory, and it is incapable of differentiating *Entamoeba histolytica* from non-pathogenic species such as *Entamoeba dispar* or *Entamoeba moshkovskii* [10]. Currently, specific and sensitive diagnostic methods to detect *Entamoeba histolytica* in stools include stool antigen detection and polymerase chain reaction (PCR) techniques based on amplification of the target parasite RNA and DNA [4]. Unfortunately, in low-resource countries, where the incidence of amoebiasis is highest, these tests are not routinely used and are not widely available for the diagnosis. Unfortunately, in our hospital blood and fecal cultures are also not available. The characteristics of stool, such as whether it is watery, bloody, mucoid, purulent or bilious, are important for classifying the etiology of acute diarrhea as non-inflammatory (mostly due to a viral infection) or inflammatory (mostly due to an invasive or toxin-producing bacterial infection) [11]. However, stool’s macroscopic characteristics are misleading. Microscopic evaluation of fecal samples is an inexpensive, rapid and highly accurate method of diagnosing in developing countries.

In our patient, amoebic dysentery was initially treated by iv Metronidazole due to the poor clinical conditions with impossibility of oral administration. Metronidazole has been used for the treatment of gastrointestinal infections for >50 years [12]. Metronidazole is considered to be a cost-effective drug because of its low cost, good activity against pathogenic anaerobic bacteria and protozoa, favorable pharmacokinetic and pharmacodynamic properties and minor adverse effects. However, several mechanisms of resistance to Metronidazole in anaerobic bacteria have been proposed. These mechanisms differ among organisms, but the primary basis for resistance is decreased uptake of the drug or altered reduction efficiency [12]. Moreover, the normal microflora serve as a reservoir of antibiotic-resistance determinants, where some dissemination of resistance can occur. The impact of Metronidazole on the normal microflora varies depending on the body site involved. In addition to Metronidazole, the use of fluids was of key importance to restore hydration and prevent dehydration. The management of dehydration in malnourished children is different from that recommended in well-nourished children, as they need special alternative fluids to avoid electrolyte imbalances and fluid overload [13]. In this case, rehydration therapy included both bolus of 1/2 normal saline + 1/2 dextrose 5% and oral ReSoMal alternated with F75.

Upon arrival, empiric antibiotic therapy with Ampicillin and Gentamicin was started due to the presence of SAM. In fact, WHO recommends antibiotic treatment in children with SAM as they have an increased risk of severe infections, including bacteremia [3]. This suggests that children with SAM are severely immunological impaired; however, the precise underlying mechanisms are still unclear.

The choice of antimicrobial drug in diarrheal disease is challenging due to the broad pattern of possible pathogens according to age and local epidemiology. The human gut is full of diverse bacteria, many of which harbor resistances against a broad range of antibiotics [14]. As the gut microbiota develops and evolves throughout life, so does the resistome. Though antibiotic use (and misuse) invariably contributes to antimicrobial resistance and the composition of the resistome, there are other factors that play a role as well (i.e., diet, infections, animals and the environment) [14]. SAM children have alterations in gut microbiota, including increased *Proteobacteria* and decreased *Bacteroides* levels [15]. Additionally, the gut microbiota of SAM children exhibit lower relative diversity compared with healthy children [15]. Although stool culture or PCR would be warranted to guide therapy, they were unavailable in our Unit. Therefore, after the identification of uncountable leukocytes and mucous in stool microscopy (i.e., findings compatible with enteric bacterial co-infection), antibiotic therapy was switched to Ceftriaxone iv. This broad-spectrum antibiotic is effective against *Shigella*, *Yersinia* and gram-negative *Enterobacteria* and it is well-tolerated in children [16]. However, since the clinical picture did not improve in the following 48 h, Ciprofloxacin iv was also added. Fluoroquinolones are effective against a wide variety of enteric infections (i.e., shigellosis, salmonellosis, cholera and *Campylobacter infections*) [17]. Although Ciprofloxacin is not routinely recommended in pediatric age, its use is suggested in presence of antimicrobial resistance [18]. Increase in antimicrobial resistance has also been observed in developing countries [19]. Knowledge as well as control of this phenomenon would be fundamental to direct empiric antibiotic therapy, but there are a lack of adequate epidemiological studies to identify local pattern of agents and antimicrobial resistance in low-resource countries.

Amoebiasis is responsible for diarrheal diseases, causing significant morbidity and mortality. Public health efforts should be directed towards its control, and better diagnostic methods should be employed for distinguishing between pathogenic and non-pathogenic species of *Entamoeba* [20]. The goal is to eradicate amoebiasis from the planet, but the parasitic life of *E. histolytica* is ancient and complex and will likely continue to evolve with humans [21].

## 4. Conclusions

This case highlights that in low-income countries, amoebiasis, especially in children with SAM, may result in life-threatening complications such as hypovolemic shock and sepsis. Although stool microscopy remains the most-used diagnostic test in low-resource settings, a novel inexpensive, easy-to-use and rapid diagnostic test would be warranted to reach a microbiological diagnosis and guide clinical decision. Metronidazole represents the mainstay treatment of amoebiasis; however, broad-spectrum empiric antibiotic therapy is required in children with SAM and when a bacterial co-infection is suspected. Further studies will be necessary to identify the patterns of antimicrobial resistance in order to appropriately manage these complicated cases.

## Figures and Tables

**Table 1:** Blood examinations at admission and during hospitalization.

	Day 1	Day 2	Day 3	Day 7
CBC				
WBC, cells/μL	32,900	25,100	22,200	17,300
Gran, cells/μL	19,200	15,600	11,600	5800
Lym, cells/μL	11,100	7400	8800	9900
Hb, g/dL	11.8	10.3	9.1	10.7
PLT, cells/μL	546,000	414,000	400,000	447,000
Blood gas				
pH	7.31	7.23	7.38	
pCO_2_, mmHg	16	31	26	
Na^+^, mmol/L	132	140	141	
K^+^, mmol/L	2.7	2.4	3.5	
Ca^2+^, mmol/L	1.22	1.37	1.29	
Glucose, mg/dL	112	114	114	
Lactate, mmol/L	1.7	0.5	0.7	
HCO^3−^, mmol/L	8.1	13.0	15.4	
BE, mmol/L	−18.2	−14.6	−9.7	

CBC, complete blood count; WBC, white blood cells; Gran, granulocytes; Lym, lymphocytes; PLT, platelet count; BE, base excess.

## Data Availability

All the available data are reported in the case presentation.
